# Pulmonary Arterial Hypertension in Systemic Lupus Erythematosus: Current Status and Future Direction

**DOI:** 10.1155/2012/854941

**Published:** 2012-03-22

**Authors:** Atiya Dhala

**Affiliations:** ^1^Department of Medicine, North Bronx Healthcare Network, Jacobi Medical Center and North Central Bronx Hospital, 3424 Kossuth Avenue, Room 9C-01, Bronx, NY 10467, USA; ^2^Department of Medicine, Albert Einstein College of Medicine, 1300 Morris Park Avenue, Bronx, NY 10461, USA

## Abstract

Pulmonary arterial hypertension (PAH) is commonly associated with connective tissue diseases (CTDs) including systemic sclerosis and systemic lupus erythematosus (SLE). The prevalence of PAH in SLE is estimated to be 0.5% to 17.5%. The pathophysiology of PAH involves multiple mechanisms from vasculitis and *in-situ* thrombosis to interstitial pulmonary fibrosis which increases pulmonary vascular resistance, potentially leading to right heart failure. Immune and inflammatory mechanisms may play a significant role in the pathogenesis or progression of PAH in patients with CTDs, establishing a role for anti-inflammatory and immunosuppressive therapies. The leading predictors of PAH in SLE are Raynaud phenomenon, anti-U1RNP antibody, and anticardiolipin antibody positivity. The first-line of diagnostic testing for patients with suspected SLE-associated PAH (SLE-aPAH) involves obtaining a Doppler echocardiogram. Once the diagnosis is confirmed by right heart catheterization, SLE-aPAH patients are generally treated with oxygen, anticoagulants, and vasodilators. Although the prognosis and therapeutic responsiveness of these patients have improved with the addition of intensive immunosuppressive therapies, these treatments are still largely unproven. Recent data put the one-year survival rate for SLE-aPAH patients at 94%. Pregnant women are most at risk of dying due to undiagnosed SLE-aPAH, and screening should be considered essential in this population.

## 1. Introduction

Pulmonary arterial hypertension (PAH) is a complex and devastating disease. PAH is defined as an increase in mean pulmonary arterial pressure (mPAP) ≥25 mmHg at rest, pulmonary artery wedge pressure (PAWP), or left ventricular end diastolic pressure ≤15 mmHg and increased pulmonary vascular resistance (PVR) [1]. PAH can be idiopathic (IPAH), heritable, drug, or toxin induced or associated with human immunodeficiency virus infection, portal hypertension, congenital heart diseases, schistosomiasis, or chronic hemolytic anemia. It can also be associated with varied connective tissue diseases (CTDs) such as systemic sclerosis (SSc), systemic lupus erythematosus (SLE), rheumatoid arthritis (RA), or mixed connective tissue disease (MCTD). These PAH-associated conditions are categorized in the World Health Organization (WHO) Group 1 PAH classification [1, 2].

While the pathophysiologic pathways linking PAH to SLE have not been adequately explored, this paper will address the key research findings and available data on this subject, as derived from an extensive literature review. PAH disease progression is characterized by narrowing of the pulmonary arterial bed due to extensive endothelial, adventitial and smooth muscle dysfunction. Genetic, environmental, and other predisposing conditions, including vasodilator and vasoconstrictor imbalance, inflammatory and uncontrolled immune response, and an imbalance between proliferation and apoptosis [3, 4], lead to constrained blood flow, potentially resulting in increased pulmonary vascular resistance. Patients with unrecognized PAH or those who are not yet treated progress to right ventricular dilatation and failure, which can ultimately lead to death.

Recent intensive immunosuppressive and vasodilator therapies have shown a lot of promise in treating SLE-associated PAH (SLE-aPAH). Recent data reveal that one-year survival rate was notably higher (at 94%) in SLE-aPAH patients when compared to that for SSc-aPAH patients (at 82%) [5, 6]. The hospitalization rates were also significantly lower in SLE-aPAH patients. Although the prognosis and therapeutic responsiveness of these patients have improved relative to the better understood SSc-associated PAH patients (SSc-aPAH), these therapies are still unproven and require further study.

## 2. Prevalence and Demographics

The prevalence of all PAH has been estimated at 15 cases per million (adults) according to the national French registry [7]. Studies from France and Scotland estimated the prevalence of CTD-associated PAH (CTD-aPAH) to be 2.3 and 10 cases per million, respectively, within their general population [7, 8]. The prevalence of PAH in SLE is estimated to be 0.5% to 43% in some older studies [9–12] and 0.5% to 17.5% in two newer French studies [13, 14]. The estimated prevalence range is wide, caused by multiple factors such as varied population groups, lack of a uniform PAH definition, and different diagnostic approaches (echocardiogram versus right heart catheterization (RHC)) [9–14]. In a large community-based lupus cohort from the United Kingdom (*n* = 288), the prevalence of SLE-aPAH was 4.2%. However, the UK study used echocardiogram, which tends to yield estimated systolic pulmonary artery pressures that can differ significantly from the “gold standard”, RHC [9].

The Registry to Evaluate Early and Long-term Pulmonary Arterial Hypertension Disease Management (REVEAL) is a 54-center longitudinal US based registry for patients with PAH. It has the largest cohort of patients (*n* = 2, 967) with PAH confirmed by RHC. The registry included 641 patients with CTD-aPAH, of which 110 patients had SLE-aPAH, including approximately 15 patients with newly diagnosed SLE-aPAH.  Table 1 provides a comparative analysis of demographic and diagnostic features of the IPAH, CTD-aPAH, SLE-aPAH, and SSc-aPAH patients observed in the registry. Patients with SLE-aPAH were younger compared to other CTD-aPAH patients. Both SLE-aPAH and CTD-aPAH patient groups were comprised predominantly of women who had similar body mass indices.

Cohort studies, other than REVEAL, have similarly confirmed the SLE-aPAH patients' demographics: patients are predominantly females of child-bearing age, from 18 to 40 years, with a female to male ratio of 10 : 1. The majority of patients with SSc-aPAH in the REVEAL cohort were white (84%), compared with only 37% of SLE-aPAH patients. Approximately one-third of patients with SSc-aPAH and MCTD-aPAH were reported to suffer from Raynaud phenomenon, compared with 14% of patients with SLE-aPAH (*P* < 0.0001). Although other studies have estimated the prevalence of Raynaud phenomenon in SSc and SLE to be as high as 90% and 45%, respectively, the registry revealed low numbers for both SLE and SSc due to underreporting of this data in the REVEAL registry [6, 11, 15–18].

Two large cohort studies have examined the differences in treatment of SLE-aPAH versus SSc-aPAH. SLE-aPAH patients were more likely to receive immunosuppressive therapies in both US and UK cohorts. In the REVEAL cohort (US), 22% of SLE-aPAH patients received immunosuppressive therapy versus 6.8% of SSc-aPAH patients. Due to the different therapeutic approaches, nearly four times as many UK based cohort patients with SLE-aPAH received immunosuppressive therapy [5, 6].

## 3. Pathobiology of Systemic Lupus Erythematosus-Associated Pulmonary Hypertension

**Figure 1:**
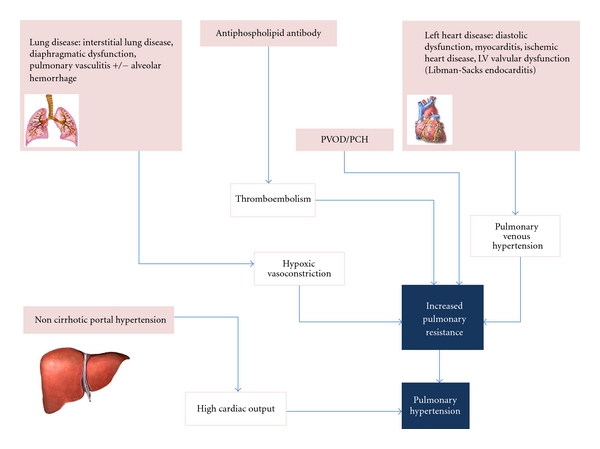
Pathophysiology of pulmonary hypertension in systemic lupus erythematosus. Pulmonary venoocclusive disease (PVOD); pulmonary capillary hemangiomatosus (PCH); left ventricle (LV).

Although a causal relationship between SLE and PH has not been established, the various elements of SLE, from vasculitis and *in-situ* thrombosis to interstitial pulmonary fibrosis, can lead to endothelial and smooth muscle proliferation and damage of the pulmonary vasculature resulting in PH [16–18, 28]. Increased pulmonary vascular resistance may result from multiple mechanisms in patients with SLE-aPAH, including hypoxia due to lung disease (hypoxic vasoconstriction), pulmonary venous hypertension due to left heart disease, antiphospholipid antibody predisposing to *in-situ* thrombosis or acute/chronic thromboemboli, high output state from non cirrhotic portal hypertension, and pulmonary venoocclusive disease (PVOD)/pulmonary capillary hemangiomatosis (PCH) [29–35] (refer to Figure 1).

Autopsy findings in multiple reports suggest multifactorial mechanisms for SLE-aPAH. Vascular pathologic findings in patients with SLE-aPAH are similar to those in patients with IPAH, including the plexiform lesions, muscular hypertrophy, and intimal proliferation [36]. In addition, studies have shown an imbalance between vasoconstrictors and vasodilators in SLE-aPAH, with higher levels of endothelin-1 [37] and thromboxane A2 and an inhibition of prostacyclin production by endothelial cells. It should be noted that these imbalances (elevation of thromboxane A2 and inhibition of prostacyclin) have not been shown specifically in SLE patients and are extrapolated from IPAH and SSc data. Another key mechanism involves immunoglobulin and complement deposition in the arterial walls [38, 39]. Tables 2 and 3 summarize the pathology and the key causative mechanisms in pulmonary arterial hypertension due to systemic lupus erythematosus [40–64].

Immune and inflammatory mechanisms may play a significant role in the pathogenesis or progression of PAH, especially in patients with connective tissue diseases, establishing a role for anti-inflammatory/immunosuppressive therapy. The inflammatory hypothesis in PAH has been validated in multiple studies, due to the finding of infiltration of macrophages and lymphocytes in the plexiform lesions. Similarly, the finding of IgG and complement in the pulmonary artery walls lends support to the immune hypothesis. Furthermore, in support of the immune mechanism, researchers have found elevated serum levels of proinflammatory cytokines and overexpression of growth factors in diseased pulmonary arteries of severe PAH patients [4, 40, 49, 52]. 

**Figure 2:**
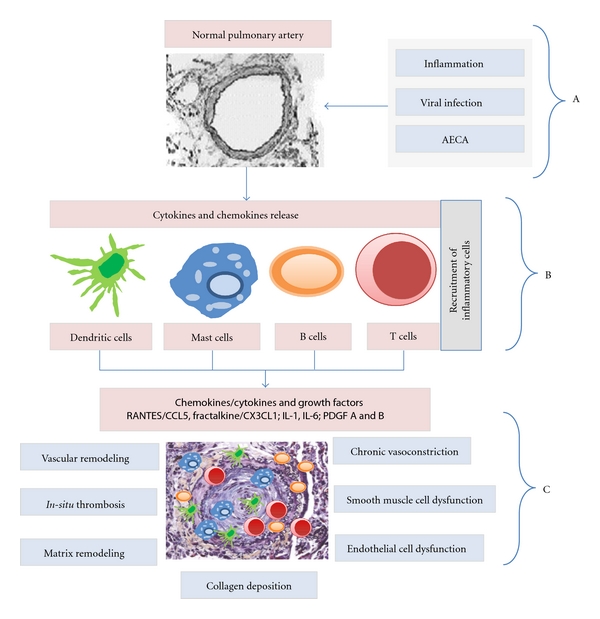
Role of inflammation and Dysregulated immune response in the development of PAH in SLE. (A) Viral infection, AECA, and other agents damage the normal pulmonary endothelium. (B) Increase in chemokine/cytokine concentrations as a result of endothelial injury, leading to recruitment of dendritic cells, mast cells, B cells, and T cells. (C) Infiltration of the small, and medium-sized pulmonary arteries by the dendritic cells, mast cells, B cells and T cells, resulting in dysregulated angiogenesis. AECA: antiendothelial cell antibodies; RANTES: regulated upon activation, normal T cell expressed and secreted; CCL5: chemokine Ligand 5; CX3CLI: chemokine Ligand 1 [Fractalkine]; IL-1: interleukin-1; IL-6: interleukin-6; PDGF: platelet derived growth factor.

Figure 2 is a diagrammatic representation of the role of inflammation and dysregulated immune response in the development of PAH in SLE [52].

## 4. Clinical Features

The most common presenting symptoms of SLE-aPAH are dyspnea, chest pain, dry cough, and fatigue. The onset of PH in patients with SLE does not correlate with disease duration or the degree of extrapulmonary manifestations of the illness, and patients may present with PH in advance of their diagnosis of SLE. Physical findings of elevated jugular venous pressure, fixed S2, murmurs of tricuspid or pulmonic insufficiency, liver enlargement ascites, and lower extremity edema occur as a consequence of right ventricular strain, enlargement, or failure.

The study by Lian et al. [65] examines the predictors contributing to SLE-aPAH as shown in Table 4. By using univariate and multivariate regression models, the authors identified the leading predictors of PAH in SLE to be Raynaud phenomenon, anti-U1 RNP antibody, anticardiolipin antibody positivity, and serositis (statistically significant in the univariate regression model only), noted as § in the table. Echocardiography to evaluate pulmonary artery pressure and right heart function is recommended in SLE patients with these leading independent predictors. Additionally, patients with SLE-aPAH tend to have a high SLE disease activity index score.

The extrapulmonary symptom of Raynaud phenomenon, one of the major predictors of PAH in SLE, is present in 75% of patients with SLE-aPAH and in 10%–45% of all patients with SLE [11, 17, 18].

Pleural effusions are uncommon in pulmonary arterial hypertension. In a study of 89 patients with PAH associated with CTD, Luo et al. [66], demonstrated that 39.3% of the patients had trace to small and bilateral pleural effusions including 37.5% of the patients (6 out of 16) with SLE-aPAH. When compared with the patients without pleural effusions, the patients with pleural effusions had significantly higher mean right atrial pressures and lower cardiac indices.

Exercise intolerance is common in patients with SLE. However, the assessment of these patients can be confusing and difficult because the intolerance could also be attributed to other concomitant conditions such as physical deconditioning, arthritis/arthralgias, obesity, myopathy or neuropathy [39].

## 5. Antiphospholipid Syndrome

Antiphospholipid syndrome (APS), defined as the continuous presence of antiphospholipid antibody (aPL) with arterial, venous, or small vessel thrombosis, with or without recurrent pregnancy losses, can occur in association with SLE. The high prevalence of antiphospholipid antibodies in SLE-aPAH patients is well known, occurring in 83% of patients with SLE-aPAH and in 30% to 50% of patients with SLE without PAH, compared to 7% in patients with systemic sclerosis. These antibodies have been reported in 10% to 15% of IPAH patients who may be at risk for developing an underlying CTD, such as SLE, later on in the disease course.

Pathogenic aPLs activate the endothelial cells, monocytes, and platelets leading to a prothrombotic state. Patients with these antibodies are more susceptible to developing thrombotic arteriopathy and therefore require a careful assessment for chronic thromboembolic pulmonary hypertension (CTEPH). Increased levels of circulating endothelin-1 have been reported in patients with aPL, possibly contributing to vasoconstriction and PAH [47–51]. Patients with APS and SLE with high levels of aPL also have increased prevalence of valvular disease (Libman-Sacks endocarditis) which can contribute to pulmonary venous hypertension.

## 6. Diagnosis

PAH may be suspected due to findings on routine chest radiography and/or 12-lead electrocardiogram, obtained in the evaluation of dyspnea. Computerized tomography of the lung to rule out pulmonary parenchymal abnormalities is not recommended in the absence of abnormalities on physical exam, routine chest radiograph, or pulmonary function testing. The first-line of diagnostic testing for patients with suspected SLE-aPAH involves obtaining a Doppler echocardiogram to look for elevations in estimated pulmonary artery pressure and/or tricuspid valve insufficiency. The estimation of pulmonary artery pressure (PAP) by Doppler echocardiography (DE) does not necessarily correlate with the measurement of PAP obtained directly by RHC [67–69]. During the RHC, vasodilator agents such as nitric oxide, epoprostenol or adenosine may be used to identify vasoreactivity. DE may, in some instances, be used in lieu of the RHC to follow patients while on therapy; right ventricular parameters such as tricuspid annular plane systolic excursion (TAPSE) and right ventricular fractional area change could be useful indices for evaluating right ventricular function. RHC is required to confirm the diagnosis and assess the severity of PH and also to provide definitive assessment while on therapy. Most patients in this SLE-aPAH population are not vasoreactive and calcium channel blocker therapy has not proven to be beneficial. The mPAP of >30 mmHg during exercise is no longer considered to be part of the definition of PAH as the normal baseline mPAP for exercise had not been established.

Other studies to consider as part of the evaluation for secondary causes of PH, even in a patient with known SLE, include polysomnography to evaluate for sleep disordered breathing, testing for human immunodeficiency virus, hepatitis serology, pulmonary function tests (the finding of an isolated defect in diffusing capacity for carbon monoxide on lung function testing may be an early predictor of SLE-aPAH [70]), and ventilation perfusion scan to evaluate for acute or chronic thromboemboli.

## 7. Treatment

SLE-aPAH patients are generally treated with therapies such as oxygen, anticoagulants, calcium channel blockers, and vasodilators, similar to the therapeutic interventions for patients in WHO Group I. However, no single therapeutic regimen has been shown to be fully effective in treating SLE-aPAH. The vasodilators employed are selective and nonselective endothelin receptor antagonists (ETRAs), phosphodiesterase-5-inhibitors (PDE-5-I), and oral, inhaled, subcutaneous, or intravenous prostanoids [1].

The key findings in the vasodilator trials (summarized in Table 5) show improvement in exercise capacity, hemodynamic parameters, New York Heart Association Functional Class, increase in time to clinical worsening, and a trend towards improved quality of life in CTD-aPAH patients. The number of patients with SLE-aPAH in these trials was small, and most studies did not perform subgroup analysis for SLE-aPAH patients. As a result, no definitive conclusion can be drawn for this subgroup of patients. However, one study by Badesch et al., on behalf of the SUPER study group (SUPER 1), performed a posthoc analysis to study the efficacy of sildenafil on CTD-aPAH patients (*n* = 278) of which 23% had SLE-aPAH. This double-blinded study showed significant improvement in pulmonary hemodynamics, exercise capacity, and WHO functional class with 20 mgs of Sildenafil over a 12-week period [22].

As discussed earlier, patients with aPLs are more susceptible to *in-situ* thrombosis and thrombotic arteriopathy and should be screened for chronic thromboembolic pulmonary hypertension (CTEPH). Once diagnosed, the CTEPH patients require different modalities of treatment [71–75].

Another important condition seen in patients with PH associated with SLE is mitral and aortic valvular pathology (referred to as Libman-Sacks endocarditis) causing regurgitation and leading to pulmonary venous hypertension. The precise incidence has not been determined and effective treatment is unavailable [76–81].

As discussed in the pathology section, SLE-aPAH results from sustained pulmonary vasoconstriction leading to luminal obliteration of small and medium-sized pulmonary arteries, due to the formation of plexiform lesions and *in-situ* thrombosis. In addition, inflammatory and dysregulated immune components play a major role in the pathogenesis of PAH in SLE leading to therapy with anti-inflammatory glucocorticoids and immunosuppressive therapies, a subject of on-going investigation [19–21, 23–27, 82–84].

Table 5 delineates the various treatment modalities and their respective outcomes in patients with SLE-aPAH. In the studies summarized in the table, all patients had RHC for diagnosis of PAH. It should be noted that the immunosuppressive therapy trials to date have been small nonrandomized, observational, retrospective, uncontrolled studies (with historical controls) and case reports, whereas the vasodilator treatment studies are mostly randomized controlled studies with a small number of SLE-aPAH patients. Additional comprehensive and controlled trials are needed to test the effectiveness of immunosuppressive therapies in the SLE-aPAH patients.

Intensive immunosuppressive therapy (IIT) is defined as an intravenous (IV) bolus of cyclophosphamide 500–600 mg/m^2^ monthly for 3–6 months in addition to oral glucocorticoids 0.5–1 mg/kg/day for 4 weeks followed by a slow taper. In the three studies [19–21] highlighted in the table, variations of above mentioned doses and time periods of administration of cyclophosphamide and oral or intravenous glucocorticoids were used.

## 8. Other Therapies

Atrial septostomy and lung or heart-lung transplantation may be an option for some patients with SLE-aPAH who have failed maximized medical therapy and continue to have disease progression (acceptance for transplant maybe predicated upon quiescence of other systemic manifestations of SLE) [85]. IPAH patients have a better prognosis than SLE-aPAH patients. Most patients with SLE-aPAH are women of a child bearing age, and due to the high maternal mortality in this group, screening for PH in pregnant mothers is recommended [86–90].

## 9. Survival

The one- and three-year survival rates for SLE-aPAH are 78% and 74% respectively [2, 5]. While the one-year survival rate of SSc-aPAH patients is similar to that of SLE-aPAH patients, the three-year survival rate is much lower at 47%. The REVEAL cohort of patients with SLE-aPAH had a one-year survival rate of 94% [6]. The advanced therapy including immunosuppression given to SLE-aPAH patients early in the course of disease may account for the improved survival rates, despite similar abnormalities in baseline pulmonary hemodynamics in both patient groups. However, if other respiratory disorders coexist with PH, the prognosis is similar to that of patients with SSc-aPAH.

Quadrelli et al. [91] examined 90 SLE necropsies and found 97.8% to have some pleuropulmonary involvement but not necessarily related to SLE. The most frequent lung finding was bacterial bronchopneumonia (a contributing cause of death) in 90% followed by pleuritis in 88%. Four out of 90 patients had findings of pulmonary hypertension (4.4%). In another study [92], pulmonary arterial hypertension was the third most common cause of death, after infection and lupus manifestations other than renal involvement. The patients in the latter study were on higher doses of corticosteroids preceding their death.

## 10. Conclusions

Connective tissue disease-associated PAH has historically had a poor prognosis with a one-year survival rate of 45% in patients with SSc-aPAH. Recent survival rates of all CTD-aPAH have improved, in part due to the advances in therapies, although these modalities require further study. To date, most of the research has focused on SSc-aPAH, leaving insufficient data on the other CTD-aPAH. Jais, Sanchez, and colleagues [19–21, 82] have studied SLE-aPAH and the effect of intensive immunosuppressive therapy on the survival rates. The linkage between intensive immunosuppressive therapy and improved survival rates is not yet conclusive due to the paucity of randomized placebo-controlled studies. These studies are difficult to conduct because there are few patients with this disease who are not already on therapy. However, patients should be treated aggressively with immunosuppressive and anti-inflammatory therapies, coupled with vasodilator therapy due to progression of disease. In certain cases, an initial combination of intensive immunosuppressive and vasodilator therapies may be used [93, 94].

To date, there have not been consensus recommendations for screening for PH in patients with SLE. However, young women of child bearing age are most at risk of dying due to undiagnosed SLE-aPAH during pregnancy, delivery, and post partum. Therefore, screening should be considered essential in this population. Patients with anti-U1 RNP antibody, anticardiolipin antibody, and Raynaud phenomenon should also be seriously considered for screening, given the high correlation between these predictors and PH.

## Figures and Tables

**Table 1:** REVEAL registry demographic and diagnostic comparison.

	IPAH	CTD	SLE-aPAH	SSc-aPAH
Total # of patients	1251	641	110	399
Patients newly diagnosed at enrollment (%)	14	15	14	16
Age (years)	50.1 ± 17.5	57.1 ± 13.7	45.5 ± 11.9	61.8 ± 11.1
Sex, (#)				
Female	987	578	104	353
Male	264	63	6	46
Race (%)				
White	74.8	71.8	37.4	83.9
African-American	11.7	16.5	31.8	10.9
Hispanic	8.3	7.5	17.8	3.6
Other	5.2	4.2	13.1	1.6
Raynaud phenomenon (%)	1.4	26.5	13.6	32.6
Renal insufficiency (%)	3.9	6.9	4.6	8.7
Time between diagnostic RHC and enrollment (months)	41.1 ± 44.1	27.2 ± 29.9	34.4 ± 39.1	24.2 ± 24.1
BNP (pg/mL)	245.6 ± 427.2	432.8 ± 789.1	263.8 ± 338.8	552.2 ± 977.8
DLCO (%)	63.6 ± 22.1	44.9 + 18	53.3 ± 19.5	41.2 ± 16.3
Immunosuppressive therapy (%)	1.3	11.9	22	6.8
Alive at 1 year (%)	93	86	94	82

**Table 2:** Pathology of systemic lupus erythematosus associated pulmonary hypertension.

Pathological changes in arteries, arterioles and veins	
(i) Medial hypertrophy	
(ii) Chronic intimal fibrosis	
(iii) Periadventitial fibrosis	
(iv) Alteration of elastic laminae	
(v) Necrotizing fibrinoid arteriopathy	
(vi) Aneurysmal dilatation and plexiform lesions	

Pathological changes in Thrombotic Arteriopathy	

(i) Intimal eccentric fibrous thickening	
(ii) Luminal occlusion with recanalization	
(iii) Plexiform lesions coexistent with intimal thrombotic lesions in some arteries	
(iv) Concentric laminar intimal fibrosis not present	

**Table 3:** Key causative mechanisms of PAH in systemic lupus erythematosus.

Mechanisms similar to IPAH patients	
(i) Overactivation of transcription factors (hypoxia inducible factor-1 alpha and Nuclear Factor of activated T lymphocytes)	
(ii) Decreased expression of certain voltage gated potassium channels	
(iii) *De novo* expression of the antiapoptotic proteins	

Mechanisms involving inflammation and autoimmunity	

(i) Chronic inflammation caused by viral infections and autoimmune diseases, leading to the migration of monocytes, neutrophils, mast cells, and dendritic cells to the structurally damaged pulmonary artery	
(ii) Invasion of the elastic lamina, stimulating the release of chemokines, cytokines and growth factors	
(iii) Resultant vascular remodeling, collagen deposition, and uninhibited proliferation of endothelial cell	

Immune dysregulation mechanism	

(i) Decreased percentage of CD4^+^/CD25^+^ T cells, diminished regulation by regulatory T cells and B cells, and stimulated signals to B cells	

Pathology involving autoantibodies	

Antiendothelial cell antibodies (AECA)	
(i) AECA prevalence ranges from 15% to 80%	
(ii) AECA levels are increased in active SLE, in particular in patients with nephritis, PH and vascular injuries.	
(iii) AECA enhances release of endothelin-1	
(iv) Binding of AECA or immune complexes may augment release of interleukin-1 (IL-1) and tumor necrosis factor-alpha (TNF-*α*)	
Antiphospholipid antibodies (aPL)	
(i) Present in 40% of patients with SLE	
(ii) aPLs activate the endothelial cells, monocytes, and platelets leading to a prothrombotic state	
Other autoantibodies in SLE-associated PAH	
(i) Antinuclear antibody (ANA) invariably present	
(ii) >25% prevalence of ribonuclear protein (RNP)	
(iii) 50% to 80% prevalence of rheumatoid factor (RF)	

**Table 4:** Possible risk factors for the development of PH in systemic lupus erythematosus.

(i) Female gender	
(ii) Isolated reduction in diffusion	
(iii) Raynaud phenomenon §	
(iv) Serositis §	
(v) Renal disease	
(vi) Digital gangrene	
(vii) Cutaneous vasculitis/livedo reticularis	
(viii) Rheumatoid factor	
(ix) Anti-U1 RNP §	
(x) Anticardiolipin antibodies §	
(xi) Antiendothelial cell antibodies	

**Table 5:** Treatment modalities and respective outcomes for SLE-aPAH patients. Mean pulmonary artery pressure (MPAP) in mmHg; pulmonary vascular resistance (PVR) in Woods units; 6 minute walk distance (6MWD) in meters; age in years; New York Heart Association Functional class (NYHA FC); Average (avg.).

Studies	Drug/design		Patients and baseline characteristics		Outcome
Intensive Immunosuppressive therapy (IIT) trials					

	IIT: IV cyclophosphamide + oral glucocorticoids + vasodilator therapy (VT)		(i) 8 patients with SLE-aPAH		IIT:
			(ii) MPAP = 39.5 ± 9.2		(i) Significantly decreased MPAP
			(iii) PVR = 8.75 ± 5.43		(ii) Tended to decrease PVR
Miyamichi-Yamamoto et al. [19]			(iv) NYHA FC = I, II, III		(iii) Normalized hemodynamics in a few patients.
			(v) 6MWD = 442 ± 54		IIT + VT improved the pulmonary hemodynamics and long-term prognosis of patients with CTD-aPAH.
	Observational cohort study from a single center with historical control		(vi) Age = 42 ± 8		

	IIT: IV cyclophosphamide + glucocorticoids + VT		Rx with IIT	Rx with IIT + VT	(i) SLE-aPAH patients with less severe disease may respond to treatment with IIT.
		*N* = 13	9	V	(ii) For patients with more severe disease, VT should be started, possibly in combination with IIT.
		MPAP	48 ± 12	58 ± 10	
Jais et al. [20]		PVR	8.6 ± 3.5	14.3 ± 1.3	(iii) These retrospective and uncontrolled data need to be confirmed by randomized controlled trials.
	Retrospective, uncontrolled study	NYHA	II, III	III, IV	
		FC			
		6MWD	347 ± 80	381 ± 71	
		Age	31 ± 10	38 ± 9	

	IV cyclophosphamide ± glucocorticoids		(i) 13 patients with SLE-aPAH		(i) Of the responders [R] 62% had SLE.
			(ii) MPAP (avg.) = 54		(ii) R's had a significantly improved 6MWD and hemodynamic parameters.
Sanchez et al. [21]	Retrospective study		(iii) PVR (avg.) = 19		
			(iv) NYHA FC = II, III		(iii) R's had a better survival than non responders [NR].
			(v) 6MWD (avg.) = 370		
			(vi) Age (avg.) = 29		

Oral agents: endothelin receptor antagonists (ETRAs) and phosphodiesterase-5-inhibitors (PDE-5-I)					

	Sildenafil 20 mg, 40 mg, 80 mg		(i) 19 patients with SLE-aPAH		In patients with PAH-aCTD, sildenafil improves exercise capacity, hemodynamic parameters (at the 20 mg dose), and NYHA FC after 12 weeks of treatment.
			(ii) MPAP = 47 ± 11		
Badesch et al. [22]	12 week, double-blind study (SUPER-1)		(iii) PVR = 10.13 ± 5.45		
			(iv) NYHA FC = II, III, IV		
			(v) 6MWD = 342 ± 76		
			(vi) Age = 53 ± 15		

	Sildenafil 20 mg, 40 mg, 80 mg		(i) 19 patients with SLE		Sildenafil improves exercise capacity and hemodynamics in patients with symptomatic PAH. SLE-aPAH subgroup analysis was not done.
			(ii) MPAP = 52.75 ± 14		
Galiè et al. [23]	Double-blind placebo-controlled trial		(iii) PVR = 11.95 ± 6.29		
			(iv) NYHA FC = II, III, IV		
			(v) 6MWD = 344 ± 82		
			(vi) Age = 49 ± 15		

	Bosentan		(i) 16 patients with SLE		
			(ii) MPAP = 55 ± 16		Statistically significant improvement in exercise capacity, NYHA FC and increase in time to clinical worsening.
Rubin et al. [24]	Double-blind placebo-controlled		(iii) PVR = 12.68 ± 8.48		
	trial		(iv) NYHA FC = III, IV		
			(v) 6MWD = 330 ± 74		
			(vi) Age = 49 ± 16		

Subcutaneous, inhaled, and intravenous prostanoids					

	Subcutaneous treprostinil		(i) 25 patients with SLE		
			(ii) MPAP = 52 ± 2		Improved exercise capacity, dyspnea fatigue symptoms, hemodynamics and trend toward improved quality of life.
Oudiz et al. [25]			(iii) NYHA FC = II, III, IV		
	Double-blind placebo-controlled trial		(iv) 6MWD = 280 ± 13		
			(v) Age = 54 ± 2		

	Inhaled Iloprost		(i) 35 patients with CTD		(i) Statistically significant benefit in combined endpoint of 10% improvement in 6MWD and FC improvement and absence of clinical deterioration.
Olschewski et al. [26]			(ii) MPAP = 52.8 ± 11.5		
	Randomized placebo-controlled trial		(iii) PVR = 12.86 ± 4.88		
			(iv) NYHA FC = III, IV		
			(v) 6MWD = 332 ± 93		
			(vi) Age = 51 ± 13		(ii) No subgroup analysis done for SLE.

	Intravenous epoprostenol		(i) 6 patients with SLE		Dramatic improvement in FC and marked improvement in hemodynamics.
			(ii) MPAP = 57 ± 9		
Robbins et al. [27]	Case series		(iii) PVR = 14 ± 7		
			(iv) NYHA FC = III, IV		
			(v) Age = 26–35		
